# Emerging professional practices focusing on reducing inequity in speech-language therapy and audiology: a scoping review protocol

**DOI:** 10.1186/s13643-022-01953-0

**Published:** 2022-04-21

**Authors:** Kristen Abrahams, Rizwana Mallick, Ameer Hohlfeld, Tamzyn Suliaman, Harsha Kathard

**Affiliations:** 1 Department of Health and Rehabilitation Sciences, Division of Communication Sciences and Disorders, University of Cape Town, Cape Town, South Africa; 2 Faculty of Health Sciences, Department of Health and Rehabilitation Sciences, F45 Old Main Building, Groote Schuur Hospital, Observatory, Cape Town, 7925 South Africa; 3 South African Medical Research Council, Cochrane South Africa, Cape Town, South Africa; 4 UCT Libraries, University of Cape Town, Cape Town, South Africa; 5 Inclusive Practices Africa Research Unit, University of Cape Town, Cape Town, South Africa

**Keywords:** Equity, Speech-language therapy, Audiology, Scoping review, Protocol, Emerging professional practices

## Abstract

**Background:**

Human communication is essential for socialising, learning and working. Disabilities and social disadvantage have serious negative consequences on communication which can impact development from early life into adulthood. While speech-language therapists and audiologists (SLT/As) have an important role to play in addressing communication disability and disadvantage, services continue to be inaccessible, unaffordable and unattainable for the majority population. In order to support this large population, it is necessary to reimagine SLT/A practices in line with equity and social inclusion. Recently in the literature, there have been increasing calls for professions to reduce inequities in practice as indicated by the sustainable development goals, human rights and social inclusion approaches increasing in prominence. For the scoping review, equity is understood using the colonial matrix of power to understand how intersections of race, gender, class, disability, geography, heteronormativity and language create the context for inequity. As such, the aim of the scoping review is to address the following question: what are the emerging professional practices in SLT/A focused on reducing inequities?

**Methods:**

Following the Joanna Briggs Institute guidelines, this scoping review will focus on systematically mapping the documented emerging clinical practices in SLT/A in the literature to identify how the professions are developing equitable practices. The search will include electronic databases and grey literature including PubMed, Scopus, EbscoHost, The Cochrane Library and Dissertation Abstracts International, Education Resource Information Centre from their inception onwards. Published and unpublished literature including all evidence sources will be considered. There should be a clear focus on clinical practice addressing equity in SLT/A. There will be no language limitations for the study. The authors will endeavour translate to have abstracts of articles translated. There will be no time restrictions on date of publication of the literature.

**Discussion:**

We aim to review the current literature on emerging professional practices in relation to equity in SLT/A to identify emerging trends in clinical practice. It is our goal to provide a synthesis of emerging directions for practice, particularly to inform future practices in the Global South.

**Systematic review registration:**

Open Science Framework (osf.io/3a29w).

**Supplementary Information:**

The online version contains supplementary material available at 10.1186/s13643-022-01953-0.

## Background

Human communication across all modalities (written, gestural, verbal, visual) connect people to each other and helps us convey meaning, experiences and information for socialising, learning and working. As children grow, they begin to acquire communication skills. Initially children develop language skills, which are essential for later literacy development during their school aged years. As children enter high school, they begin to develop communication skills for academic learning. As adults, communication skills continue to develop as relationships, written skills and reading become essential to workplace practices. As speech-language therapists and audiologists (SLT/A), communication is central to our work [1].

Traditionally, SLT/A practices have focused on communication impairment—the consequences of which can lead to disability. According to the World Report on Disability, over a billion people (approximately 15%) are estimated to be living with a disability around the world [2]. In addition, there is equal concern around communication challenges in the context of social disadvantage [3]. Systemic challenges throughout the world such as poverty, language, geographic location and quality education affect opportunities for learning communication and subsequently impacts communication development.

The impact of communication disabilities and disadvantage have serious negative consequences which affect communication development from early life into adulthood. Communication difficulties evident in the early years continue to grow exponentially as children and their families try to make up the communication gap [4]. These systemic inequalities have an impact on communication development and subsequent learning and vocation. Here, we acknowledge the influence of colonisation in creating and maintaining long lasting and continuing inequalities. In his seminal book: *How Europe underdeveloped Africa*, Rodney [5] argued that colonial education was not meant to suit the colonised, rather it aimed to serve the interests of the coloniser ensuring their domination and exploitation of Africa. It is no coincidence that today, marginalised communities continue to be burdened with the effects of colonisation (and apartheid in South Africa). Such challenges are evident in both majority and minority world contexts. The stark contrast is that in majority world, the majority of the population is marginalised. As such, there is a need to focus on supporting communication in marginalised communities in both Majority and Minority World context.

In the context of the work of SLT/A, Pillay and Kathard [6] stated: “It is a truism that the biggest beneficiaries of our services, globally, are those who are of or ascribe to the minority world’s cultural capital—typically middle-class, usually white populations who speak a dominant world language like English” (pg. 195). As a result, poor, Black, indigenous language speaking individuals continue to be marginalised. We therefore consider inequity in the professions in relation to colonial matrix of power [7], i.e. capitalism, patriarchy, racism, heteronormativity, Christianity, geo-political and language. Grosfoguel [7] described the colonial matrix of power as “an organising principle involving exploitation and domination exercised in multiple dimensions of social life, from economic, sexual or gender relations to political organisations, structures of knowledge, state institutions and households.” (pg. 12). These factors will be used to analyse the ways in which equity has been described in the literature.

## Challenging SLT/A practices

Traditional SLT/A practices, influenced by the medical model, are characterised by one-on-one, individualised, institutionalised practices. In addition, populations for which SLT/A services were designed and who have benefitted the most are white, middle-class, English speaking from a dominant culture [6]. As such, for the majority of the South African population SLT/A (and majority world) services are inaccessible, unaffordable and unattainable. As a profession, we need to question how we too are continuing to contribute to an unjust, inequitable society through the ways in which we work and the populations we serve. As such, Abrahams, Kathard [8] argued that the profession of SLT is a project of coloniality—acknowledging the colonial influences on its professional practice (i.e. research, professional education and clinical practice). They argued that there is a need to question, critique and rethink our professional practices. Such critical engagement may open spaces for reimagining our work, from which the concept of developing emerging professional practices (EPPs) emerged.

As part of the decolonial shift, it is necessary to reimagine our practices in line with social inclusion, i.e. “the process of improving the terms of participation in society for people who are disadvantaged on the basis of age, sex, disability, race, ethnicity, origin, religion, or economic or other status, through enhanced opportunities, access to resources, voice and respect for rights” [9] (pg. 20). In shifting toward a model of social inclusion, there is a need to question our current practices and search for ways in which we can begin to support communication for all. The scoping review therefore seeks to understand EPPs in SLT/A, particularly addressing equity in their clinical practice.

In terms of EPP, there are two important concepts to highlight—(1) practice; and (2) emergence. Professional practice is defined as encompassing three main elements—professional education, research and clinical practice [10]. For this scoping review, the focus will specifically be on understanding one such aspect, clinical practice (discussed in more detail later). Emergence as the process of coming into existence places emphasis on something that is new, beginning to develop or becoming apparent. There is an additional element of innovation and creativity as something that is emerging which is different to what has been before. As such, for this scoping review, emerging professional practice is defined as practices (specifically clinical practices) which are borne out of traditional practice (i.e. practice that is historically informed the work of SLT/A—one-on-one therapy, individual, disability focus, institutionalised, guided by medical model) and are developing, changing, and adapting from this traditional practice. The scoping review will specifically seek to understand these innovations in practices. In addition, the review will seek to understand the driving factors that facilitated the innovations and changes in practice.

## Why is it important to do the scoping review?

The scoping review is an extension of KA’s PhD thesis [11] which focused on documenting and examining an EPP in clinical education. For this review, the focus shifts to understanding another aspect of our professional work, namely clinical practices.

The UN’s Sustainable Development Goals (SDGs) focus on the reduction in inequalities through addressing education, poverty and health [12]. There are strong links to equity and human rights as the goals focused on addressing many of the social challenges which impact health. Within the professional discourse, there has been a shifting focus to understanding the role of the SLT/A within social justice and human rights frameworks [8, 13–15]. Such a refocusing draws attention to how the professions can begin to work toward developing fair and just services—in line with equity. As professions, it is imperative that we consider how we are contributing to these goals through our work in communication. In particular, the scoping review will seek to provide insights into the emerging trends in clinical practices in the professions. It is important to identify and synthesise EPPs in the professions to begin to build an understanding of how practices are developing and shifting in relation to the diverse contexts in which people live. In addition, it is important to systematically identify and synthesise the ways in which equity has been defined, to reflect the various activities, similarities and differences, to begin to build an understanding of how EPPs can work to support the diverse needs of populations.

To date, there have been no systematic or scoping reviews in SLT/A that focused on understanding equity in clinical practice. As it is an emerging topic in SLT/A, the scoping review will focus on understanding the breadth of the literature and to map out the current practices in the professions [16]. We hope that through this scoping review that it will provide the professions with further pathways to realise equitable and inclusive clinical practices that are in line with human rights, social justice and the SDGs. This scoping review will focus on systematically mapping the documented emerging clinical practices in SLT/A in the literature to identify how the professions are developing equitable practice innovations. The overarching aim of the review will be to synthesise the characteristics of emerging professional practices in SLT/A clinical practice in relation to equity using the following objectives: (a) to synthesise the ways in which equity is defined in the professions; (b) to identify and describe innovations in practices; (c) to understand and describe the drivers for change in clinical practice and; (d) to develop a conceptual understanding of EPP based on the literature.

## Methods/design

This scoping review will follow the methodological procedures for scoping reviews as proposed by the Joanna Briggs Institute (JBI) [16]. The development of the JBI approach is underpinned by the pioneering work of Arksey and O'Malley [17] and Levac, Colquhoun [18]. The steps include identifying the research question, relevant studies, study selection, charting the data and collating, summarising and reporting the results. This scoping review protocol has been registered with the Open Science Framework (registration number: osf.io/3a29w). The protocol is being reported in alignment with the Preferred Reporting Items for Systematic Reviews and Meta-Analyses Protocols (PRISMA-P) statement [19] (see checklist in Additional file 1). The planned review will be reported according to the PRISMA Extension for scoping review [20] and PRISMA-Equity Extension [21].

## Identifying relevant studies

### Study selection criteria

Studies across all sources of evidence including primary studies, peer reviewed research studies, opinion pieces, book chapters, empirical studies, conceptual papers and grey literature will be included as the focus of the review will be on understanding the breadth of literature. Studies will be eligible for inclusion if the studies show a clear focus on clinical practice. In this context, clinical practice is defined as activities performed by a professional and the resources used to achieve such practice activities, e.g. physical, material, human, [10] in any setting (e.g. hospital, clinic, community, school, private practice) Secondly, studies should also address equity in the professions. For the study, “equity in health can be defined as the absence of disparities in health (and in its key social determinants) that are systematically associated with social advantage/disadvantage” [22] (pg. 256). We also differentiate between inequity and inequality. Braveman and Gruskin [22] drew attention to the fact that equity has a normative basis. Health inequity draws attention to health inequalities that are unfair or unjust. In essence, due to having certain characteristics (race, gender, class, location etc.), people are not given the same opportunities to access health care. As such, the scoping review will be specifically considering practices focusing on marginalised communities as defined through the lens of equity. In addition, we understand that inequity is not limited to a specific geographic location. The focus of the scoping review will be on Global South context. Here, we understand the Global South as those communities that experience exploitation, marginalisation and oppression. This understanding further includes spaces within the North, e.g. Indigenous communities in the Western societies who experience marginalisation. For example, refugee communities in the Global North. As the Global South is defined by geo-political boundaries, it is not sufficient to solely limit the search to geographic location. As such, links to the Global South will be determined during the screening process. Thirdly, literature from SLT/A will be included. There will be no language limitations for the study. The authors will endeavour translate to have abstracts of articles translated within the capacity and funding of the researchers. There will be no time restriction on the date of publication of the research.

Studies will be excluded from the review if papers do show clear intention for equity, clinical practice, and communication. While we acknowledge that the scope of practice for SLT/A includes swallowing and balance, papers that solely focus on these aspects will not be included.

### Search strategy

A general search will be conducted to assist with the following: (1) identify if a similar scoping review has already been conducted and is so when it was conducted; (2) to refine the inclusion and exclusion criteria; and (3) to determine topic viability. According to the specification of the JBI, there a three-step search strategy will be used [16]. The search will be conducted by University of Cape Town librarian (TS). The search strategy will incorporate the following steps: (1) TS will be consulted to assist with refining the research question, identify relevant databases and to develop an initial search strategy for the scoping review; (2) an initial limited search of two online databases relevant to topic, namely Scopus and EbscoHost, will be conducted. Following the initial search, an analysis of the text, particularly the title, abstracts and index terms, will be conducted to identify relevant keywords; (3) the keywords identified in the initial search will be used to conduct a second search across all of the included databases to identify relevant full text articles. The following databases will be included in the search: *PubMed, Scopus, EbscoHost* [including Academic Search Premier, Africa-wide Information, Cumulative Index to Nursing and Allied Health, Education Resources Information Center, Health Source (consumer edition)], *The Cochrane Library* [Cochrane Database of Systematic Reviews, Cochrane Central Register of Controlled Trials and Cochrane Methodology Register] and *Dissertation Abstracts International, Education Resource Information Centre.* We will not specifically look for grey literature but will not exclude it when searching the various databases. It should be noted that the reference lists of the included articles will be searched in order to identify any additional studies. The reviewers will contact the authors of primary studies for further information, if required. A draft complete search strategy for the main electronic databases is available in Additional file 2.

It should be noted that the search strategy will be iterative—as the reviewers become more familiar with the literature and evidence base, additional key words, search terms may be incorporated into the search strategy. A librarian familiar with the Health Sciences (TS) and familiar with SLT/A literature will provide input in the design and refinement of the search strategy.

### Source of evidence selection

The reviewers will use the protocol developed to guide their selection process for the sources of evidence. Endnote will be used to manage the results of the search. In addition, Rayyan [23] will be used to assist with screening process.

#### Piloting of protocol

The JBI suggest the following pilot testing framework: The study protocol will be piloted so that the document can be refined. A random sample of 25 titles/abstracts will be selected. Two reviewers (KA, RM) will screen the abstracts using the eligibility criteria and definitions. The reviewers will compare their findings and discuss any discrepancies and subsequently modification will be made to the protocol. The key changes to the protocol will be documented and explanations for why changes were made will also be noted. A full-screening process will only start once < 75% agreement between reviewers is reached.

#### Full review of sources of evidence

The review will use the following steps in selecting sources of evidence. Each of the steps will be conducted independently by two reviewers (KA, RM). Initially, article titles will be reviewed to ensure a focus on marginalised communities. If uncertainty about the article title, articles will not be eliminated until it is examined in more depth during the subsequent steps. Two independent reviewers (KA, RM) will review the titles and abstracts of the articles using the inclusion and exclusion criteria. Following which, the two reviewers will screen full text articles to determine if they meet the inclusion and exclusion criteria. Separate appendices will be developed which will provide details of the included studies and a brief description of the excluded sources including the basis for why the studies were excluded.

Two reviewers (KA, RM) will conduct the screening of the articles independently following the protocol. A consensus will need to be reached by the reviewers for any disagreements in study selections. Throughout the process, the review process will be modified accordingly. A flow chart showing details of studies included and excluded at each stage of the study selection process will be provided.

### Data extraction

The results will be presented in a descriptive summary linked to the aims and objectives of the scoping review. A draft chart will be developed to record key information for the source such as author, reference, and year of publication. This template will be used as a basis to design data extraction tool for the study. In order to ensure a comprehensive tool, the reviewers will test the extraction tool on two studies to ensure that the relevant information is extracted and captured. For the data extraction process, two reviewers (KA, RM) will independently chart the data using Microsoft Excel. Following which, the charting results will be discussed to ensure consensus. The charting process will be iterative and therefore as additional relevant information becomes evident the tool can be updated before completing the full scoping review extraction. Table 1 documents the framework that will be used for the data extraction that will be presented based on the study details, study characteristics and aims and objectives of the review.

**Table 1:** Framework for data extraction

Study details	Characteristics of study	Aims/objectives
*Citation* (incl. authors, study title, journal) *Country* (i.e. location of lead author) *Year of publication* (i.e. when was the article first published?)	*Population of interest* (e.g. Refugee, migrant etc.) *Geographical context* (i.e. where study took place) *Type of stud*y (e.g. qualitative, quantitative) *Clinical practice* (i.e. description of practice)	*Definition of equity* (e.g. human rights, naming of marginalised group) *Drivers for change* (e.g. what is pushing for change?) *Practice innovation* (i.e. how are the practices shifting from the traditional?) *Conceptual understanding of equity* (i.e. what are the key principles informing shift toward equitable practices?)

### Analysis of evidence

Descriptive and thematic analysis will be conducted by summarising, organising and reporting on emerging practices in SLT/A. Descriptive statistics will describe the nature and distribution of the included studies as informed by the data extraction tool. In addition, thematic analysis will be used to identify similarities and differences between practices and key drivers influencing EPPs. The development of the framework for the analysis of evidence will be an iterative process and therefore will continuously be adapted throughout the scoping review process. The analysis of evidence will specifically consider inequity and innovation.

#### Inequity

The Ecology of Human Performance framework [24] and the colonial matrix of power [7] will be used as guiding frameworks to understand the drivers for change in practice in the context of inequity. The Ecology of Human Performance framework [24] postulates that ecology (or context) affects human behaviour and as such behaviour cannot be understood outside of context. If the context shifts, the behaviours required to accomplish the goal will change. Using the framing, we theorise that a change in context will necessitate a change in clinical practices. As such, we will consider how the person-centred (i.e. experiences, skills and abilities) and contextual (physical, temporal, social and cultural) factors may act as drivers for change toward developing EPPs. The colonial matrix of power [7] will be used to deepen this understanding as the intersection of gender, race, class, language etc. are acknowledged as creating the context for inequity within professional practice. The outcome of the scoping review would be to identify the innovations in clinical practice.

#### Innovation

The defining of what constitutes “non-traditional”, “new” or “innovative” have not been clearly established in the SLT/A literature. According to Thomasz and Young [25] the word “non-traditional” is used frequently but there is no consensus as to a definition. The scoping review is particularly interested in understanding the innovations in clinical practice. As such, innovation will be defined as practices which are moving beyond the traditional model of practice, i.e. one-on-one, individual, institutionalised, informed by medical model. This definition will also draw on the concept of decoloniality to inform our understanding of innovation. As we have understood the profession as a project of coloniality—i.e. colonial influences have shaped and continue to shape SLT/A professional practice—decoloniality will be used as a conceptual lens to understand innovation. Coloniality of knowledge, being and power specifically—e.g. challenging coloniality of being such as the doctor-patient dynamic toward developing equal contributions from each party. A conceptual framework will be developed in order to use decoloniality to understand innovations in the professions.

### Presentation of results

The expected results will be documented using a flow diagram of the search to show how studies were identified for the final analysis. In addition, tables charting the results will also be presented documenting the information in line with the data extraction tool. We will ensure that the reporting of the scoping review is in alignment with the guidelines for reports as set out by the PRIMSA 2020 statement [26].

## Discussion

Based on our searches of the literature, this will be the first scoping review within the professions of SLT/A to conceptualise and define equity and EPPs, as well as the drivers for change that are facilitating changes in practices in line with equity. In terms of potential practical and operational issues, we acknowledge that while we will endeavour to translate abstracts of potentially relevant articles, the time and financial constraints may potentially result in relevant studies being excluded from the review. We also acknowledge that the work is considering an emerging trend in SLT/A literature and therefore the ways in which we have defined concepts seminal to the work (e.g. equity, emerging practices) may require refinement throughout the review process. This may impact the selection of paper as well as the data extraction and analysis aspects of the review. As such, we have embedded an iterative process in the design of the review to ensure these potential limitations are taken into account. We further acknowledge that sources of evidence on emerging practices within the public domain may not adequately shed light on the topic as the Global South context may not be as prominent in the available literature. Amendments made to the study protocol will be documented in the publication of the final manuscript. The final manuscript will be published in a relevant peer-reviewed journal and shared more broadly through conferences and interactions with relevant stakeholders. The scoping review will be of interest to SLT/A practitioners, particularly those working in marginalised communities within the Global South as it will provide a synthesis of clinical practice and emerging directions for practice in the future.

## Supplementary Information

**Additional file 1.** PRISMA-P-checklist.

**Additional file 2.** Search strategy.

## Data Availability

The datasets used and analysed will be available from the corresponding author on reasonable request.

## Abbreviations

SLT/A: Speech-language therapists and audiologists
EPP: Emerging professional practice
SDG: Sustainable development goals
JBI: Joanna Briggs Institute
PRISMA: Preferred Reporting Items for Systematic Reviews and Meta-Analysis
