# Supplementary material for: Emerging professional practices focusing on reducing inequity in speech-language therapy and audiology: a scoping review protocol
Source: Syst Rev. 2022 Apr 21;11:74. doi: 10.1186/s13643-022-01953-0 (PMC9022329; doi:10.1186/s13643-022-01953-0)
Supplement: Supplementary file 2 — Additional file 2. Search strategy. [file 13643_2022_1953_MOESM2_ESM.docx]

**Additional file 2 –** Draft search strategy – Scopus, EbscoHost, PubMed

*Scopus Advanced Search* (182 results)

TITLE-ABS-KEY ((("speech therapy" OR "speech therapist" OR "speech pathology" OR "speech pathologist" OR "speech language therapy" OR "speech language therapist" OR "speech language pathology" OR "speech language pathologist" OR audiology OR audiologist) AND (equit* OR inequit* OR marginal* OR underserved OR "global health" OR minority OR "human rights" OR "global engagement") AND ("clinical practice" OR "practice" OR "service delivery" OR "professional practice")))

*EbscoHost* – All databases; no date restriction (832)

(("speech therapy" OR "speech therapist" OR "speech pathology" OR "speech pathologist" OR "speech language therapy" OR "speech language therapist" OR "speech language pathology" OR "speech language pathologist" OR audiology OR audiologist) AND (equit* OR inequit* OR marginal* OR underserved OR "global health" OR minority OR "human rights" OR "global engagement") AND ("clinical practice" OR "practice" OR "service delivery" OR "professional practice"))

*PubMed* (421 results)

((((((((((((((("Speech Therapy"[Mesh]) OR ("Speech-Language Pathology"[Mesh])) OR ("Audiology"[Mesh])) OR ("Audiologists"[Mesh])) OR (speech therapy)) OR (speech therapist)) OR ("speech language therapist")) OR ("speech language pathology")) OR ("speech language pathologists")) OR ("speech pathologists")) OR ("speech pathology")) OR (audiology)) OR (audiology)) OR (audiologists)) AND (((((((((((((equity) OR (equitable)) OR (inequity)) OR (inequitable)) OR (marginalized)) OR (marginalised)) OR (marginalization)) OR (marginalisation)) OR (underserved)) OR ("global health")) OR (minority)) OR ("human rights")) OR ("global engagement"))) AND (((("clinical practice") OR (practice)) OR ("service delivery")) OR ("professional practice"))

*Cochrane* – Advanced Search (9 results)

(("speech therapy" OR "speech therapist" OR "speech pathology" OR "speech pathologist" OR "speech language therapy" OR "speech language therapist" OR "speech language pathology" OR "speech language pathologist" OR audiology OR audiologist) AND (equit* OR inequit* OR marginal* OR underserved OR "global health" OR minority OR "human rights" OR "global engagement") AND ("clinical practice" OR "practice" OR "service delivery" OR "professional practice"))
